# American Whig Review

**Published:** 1852-07

**Authors:** 


*American Whig Review.*—The July No. of this popular and ably conducted Periodical, is on our table. Independent of its political bearing, it contains a great deal of interesting matter well worth $3 per year. In the present No. the table of contents presents the following bill:—The Democratic Convention; A Letter to the Publishers of Harper's Magazine; A Ghost Story; Fiat justitia ruat alison; the Desert; The United States Consular System; Posthumous Papers of P. Y.; The Incastras or Ink Fiends; The Mystery of Time; A Search after "Democratic" Principles; Our General Review; Congressional Summary; Critical Notices; Music-Books, Frontispiece; Portrait of Thomas Corwin, Secretary of the Treasury.

*The Napoleon Dynasty, or, the History of the Bonaparte Family.*—At this time, this cannot but prove an interesting book, and we doubt not the publishers will reap a golden harvest.

